# Booster COVID-19 Vaccines for Immune-Mediated Inflammatory Disease Patients: A Systematic Review and Meta-Analysis of Efficacy and Safety

**DOI:** 10.3390/vaccines10050668

**Published:** 2022-04-22

**Authors:** Ainsley Ryan Yan Bin Lee, Shi Yin Wong, Sen Hee Tay

**Affiliations:** 1 Yong Loo Lin School of Medicine, National University of Singapore, Singapore 119228, Singapore; e0363343@u.nus.edu (A.R.Y.B.L.); e0421833@u.nus.edu (S.Y.W.); 2 Division of Rheumatology, Department of Medicine, National University Hospital, Singapore 119228, Singapore; 3 Department of Medicine, Yong Loo Lin School of Medicine, National University of Singapore, Singapore 119228, Singapore

**Keywords:** COVID-19, COVID-19 vaccines, immune-mediated inflammatory diseases, autoimmune diseases, antirheumatic agents, rituximab, seroconversion

## Abstract

Background: Seroconversion and longevity of vaccine-induced immune response is blunted in immune-mediated inflammatory disease (IMID) patients owing to immunosuppressive regimens. COVID-19 booster vaccines after a primary series have been proposed with inconclusive evidence on efficacy to date. Methods: This PROSPERO-registered systematic review (CRD42022302534) was conducted according to PRISMA guidelines. PubMed, EMBASE, CENTRAL, Web of Science, CORD-19, WHO ICTRP, and medRxiv were searched up to 28 February 2022 for eligible studies. Risk of bias was assessed using the Joanna Briggs Institute critical appraisal tools. Results: From 6647 records, 17 prospective studies were included for systematic review and 12 in meta-analysis of primary series non-responders. The risk of bias was low. Pooling 340 non-responders, a booster dose proved effective with 0.47 seroconverting (95% CI: 0.32–0.63, I2 = 82%). Rituximab therapy was associated with significant impairment, with risks of 0.25 (95% CI: 0.17–0.36, I2 = 50.7%) versus 0.81 (95% CI: 0.72–0.87, I2 = 0.0%) for those without rituximab therapy. A systematic review of antibody levels against COVID-19 showed several-fold increases across studies. Incidence of local and systemic adverse events, including disease flares, were either comparable or slightly increased after the booster dose compared to primary series. No major events such as myocarditis or death were reported. Conclusion: Our results show that booster doses are effective in eliciting seroconversion in non-responders, bolstering immunity to COVID-19. It has also not been associated with major adverse events.

## 1. Introduction

Since the declaration of COVID-19 as a pandemic of global health concern, patients with immune-mediated inflammatory diseases (IMID) have been studied as a vulnerable population with increased risks of severe infection and mortality, owing to the plethora of immunosuppressive medications IMID patients are often receiving [1,2]. Vaccinations have widely been recommended for IMID patients to afford protection against clinically-significant disease [3]. Vaccine-mediated immune responses may be blunted due to the suppressed immune system of IMID patients [4]. Furthermore, for those who seroconvert, the antibody response may be shorter lived [5].

The range of immunosuppressive medications commonly used in IMID patients can broadly be divided into conventional synthetic biologic disease-modifying anti-rheumatic drugs (DMARDs), biologic DMARDs, and targeted synthetic DMARDs with diverse effects and mechanisms leading to immunosuppression [6,7]. Therapies that result in the depletion of B cells have long been hypothesised to impair immunogenicity to vaccines, though this has been demonstrated to be significant with rituximab [8,9] and ocrelizumab [10] but not belimumab [11]. A recent systematic review demonstrated that various DMARDs, including methotrexate, mycophenolate, and Janus kinase inhibitors, impair immune responses to vaccines [12]. It is also of concern that IMID patients, owing to the systemic immune response evoked by vaccines, may lead to disease flares [13].

In previous work, it was found that seroconversion rates are depressed amongst immunocompromised patients, including IMIDs patients, following the first and second doses of COVID-19 vaccines [14,15]. This further suggests the importance of exploring additional measures such as the administration of a booster dose. To date, there have been no systematic reviews looking at the efficacy of COVID-19 vaccines booster doses in IMID patients. As institutions such as the American College of Rheumatology have moved to recommend COVID-19 booster vaccination in patients on immunomodulatory drugs, a systematic review is warranted to evaluate its efficacy and safety in IMID patients [16]. This review aims to study seroconversion rates and antibody levels post-vaccination in IMID patients. Furthermore, we seek to ascertain patient and treatment factors associated with response to booster doses in IMID patients.

## 2. Materials and Methods

### 2.1. Search Strategy

The systematic review is reported according to the Preferred Reporting Items for Systematic Reviews and Meta-Analyses (PRISMA) guidelines [17] and registered on PROSPERO at CRD42022302534.

Searches of the databases MEDLINE (via PubMed), EMBASE, Cochrane Central Register of Controlled Trials (CENTRAL), Web of Science, World Health Organisation International Clinical Trials Registry Platform, COVID-19 Open Research Dataset, and medRxiv were searched for articles published from 1 January 2021 to 28 February 2022. There was no restriction on the language of publication. The search strategy is detailed in Table S1. Additional searching of references of all included studies was performed.

### 2.2. Selection of Articles

All studies including at least five IMID patients receiving a booster dose of a COVID-19 vaccine were considered for inclusion by two researchers (A.R.Y.B.L. and S.Y.W.) with any differing opinions resolved by consultation of a third researcher (S.H.T.). Duplicates were removed using Endnote X20. A booster dose of COVID-19 vaccine was considered to be any COVID-19 vaccine after completion of a primary series of vaccination, one dose of Ad26.COV2.S or two doses of mRNA-1273, BNT162b2, AZD1222, or NVX-CoV2373. Observational studies and interventional studies were included. Single case reports or case series with fewer than five patients were excluded. Patients had to be diagnosed with IMIDs, such as rheumatoid arthritis (RA), systemic lupus erythematosus (SLE), inflammatory bowel diseases (IBD), multiple sclerosis (MS), and other diseases as defined by Kuek et al. [18].

The primary outcome of interest was the proportion of IMID patients that were tested to be seronegative after completing a primary series of COVID-19 vaccination but became seropositive after a booster dose. Seroconversion status should be determined according to a validated threshold, such as determined by the manufacturer of the assay used, which will be extracted and reported. The secondary outcome of interest was the mean or median proportion of rise in serological titre of IMID patients before and after receiving a booster dose.

Studies not adhering to the aforementioned inclusion criteria were excluded. Additionally, studies were excluded if they reported seroconversion data in a form from which the proportions, risk of seroconversion, or number of seroconverted participants could not be derived and could not be obtained after consultation of the corresponding authors.

### 2.3. Extraction of Data

Data were extracted according to a pre-determined proforma in Microsoft Excel version 16.45 by one researcher (A.R.Y.B.L.), with all key extracted data reviewed and quality-checked at the end of the data-extraction phase by the same researcher.

The study characteristics comprised of setting, primary and secondary outcomes, study design, sample size, dropout and non-response rates, and inclusion and exclusion criteria. The participant data collected comprised of age, sex, and comprehensive disease and treatment history, including immunosuppressive regimen. Intervention-related data included vaccine type and brand, number receiving each vaccine, and median or mean interval between doses. Outcome-related data comprised of assay, antibody measured, and method of measurement.

### 2.4. Risk of Bias Assessment

The methodological quality and risk of bias of the studies were assessed using the Joanna Briggs Institute (JBI) critical appraisal checklist [19]. JBI tool for case series, cohort studies and randomised-controlled studies were used to assess the studies which described the response to vaccines in patients with IMIDs [20]. Risk of bias for each study was assessed independently by two researchers (A.R.Y.B.L. and S.Y.W.) with any differing opinions resolved by consensus.

### 2.5. Analysis of Data

The generalised linear mixed effects model was used to pool the logit transformed proportions of IMID patients who achieved seroconversion after a booster dose. We assessed for and considered between-study heterogeneity as significant if the *p*-value of the Q-test was <0.10 or if the I statistic was ≥50%. Subgroup analyses were performed to see if seroconversion was influenced by age, booster vaccine administered, disease, and anti-CD20 treatment and compared with tests for subgroup differences. All analyses were conducted using R (version 4.1.0) using the meta and metafor packages and considering a two-sided *p* value of <0.05 as statistically significant. Influence analysis was further conducted using the leave-one-out method and assessment for outliers. If any studies at a high risk of bias were identified, sensitivity analysis will be performed excluding these studies.

Publication bias was assessed via visual inspection of the funnel plot for asymmetry as well as quantitatively using Egger’s test. Sensitivity analysis was conducted using the trim-and-fill method after imputing potentially missing studies.

## 3. Results

**Figure 1:**
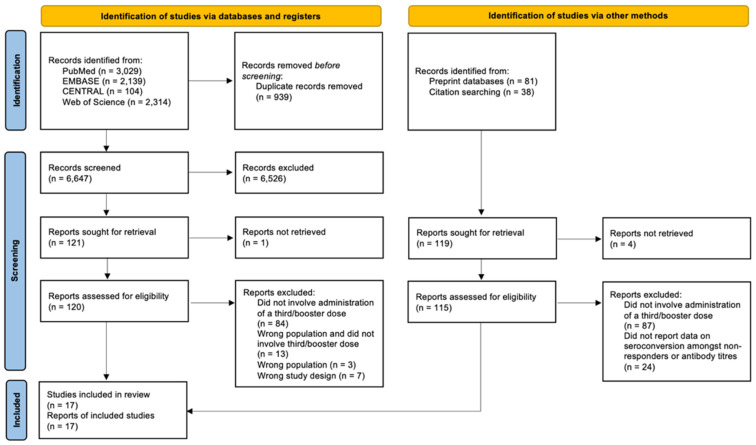
PRISMA flowchart.

From a total of 6647 records, a total of 17 prospective studies were included in this systematic review. The screening process is illustrated in the PRISMA flowchart in Figure 1. Of these studies, there were 12 observational studies [21,22,23,24,25,26,27,28,29,30,31,32], one randomised-controlled trial [33], and four case series [34,35,36,37]. The key trial characteristics of each included study are reported in Table 1, with a comparison of the characteristics of the studies included in the meta-analysis in Table S2.

COVID-19 booster vaccines administered in the studies included mRNA (Pfizer-BioNTech, BNT162b2 and Moderna, mRNA-1273) [21,22,23,24,25,26,27,28,29,30,34,35,36,37], non-replicating viral vector (AstraZeneca, AZD1222 and Janssen, Ad26.COV2.S) [21,28,29,30,34,35,36,37], protein subunit (Novavax, NVX-CoV2373) [33] and inactivated (Sinovac, CoronaVac) vaccines [35]. Bonelli et al. involved two cohorts receiving mRNA vaccines as the primary series, but one received heterologous AstraZeneca booster doses while the other received homologous mRNA boosters [29].

Most studies involved patients with diverse IMIDs. Schmiedeberg et al. [22] and Jyssum et al. [24] included only patients with RA while Felten et al. [36] included mostly RA patients and one patient with stiff-person syndrome. Schell et al. [23] included only IBD patients, and Kant et al. [37] and Speer et al. [25] only antineutrophil cytoplasmic antibody (ANCA) vasculitis patients. Achtnichts et al. [30] included patients with only MS.

### 3.1. Risk of Bias Assessment

Risk of bias assessment of all included studies was performed using the JBI critical appraisal checklists as presented in Tables S3–S5. Overall, no significant risk of bias was found.

### 3.2. Seroconversion in Non-Responders Elicited Following Booster Dose

**Figure 2:**
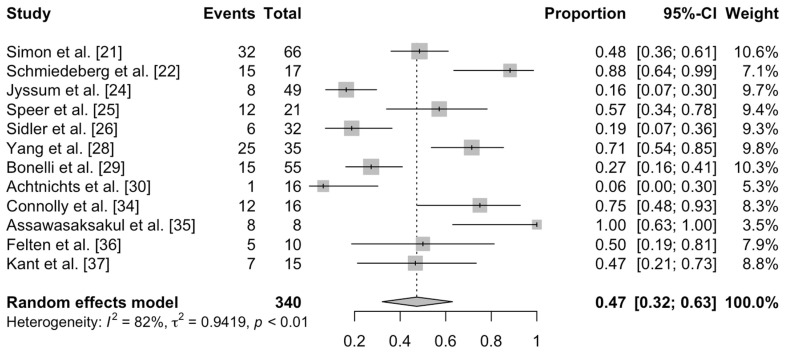
Rate of seroconversion after administration of a booster dose in non-responders to a primary series of COVID-19 vaccination.

A total of 12 studies reporting seroconversion rates in non-responders to a primary series of COVID-19 vaccination were included for meta-analysis, pooling a total of 340 non-responders after the primary series (Figure 2). The overall seroconversion risk is 0.47 (95% CI: 0.32–0.63) with the random-effects model. The overall heterogeneity was notable (I^2^ = 82%) thus showing that while booster doses could elicit seroconversion, especially-vulnerable subgroups who respond poorer would require further attention.

We undertook further subgroup analyses per protocol according to key disease and treatment factors (Table 2). Subgrouping by patients who were on anti-CD20 therapies (rituximab or ocrelizumab) versus patients on non-anti-CD20 therapies yielded significant subgroup differences (*p*-value < 0.0001). Patients on anti-CD20 therapy showed markedly-depressed seroconversion rates of 0.25 (95% CI: 0.17–0.36) compared to those without, exhibiting a seroconversion rate of 0.81 (95% CI: 0.72–0.87). Most heterogeneity was eliminated amongst each subgroup with non-anti-CD20 patients (I^2^ = 0.0%) while those on anti-CD20s showed reduced heterogeneity (I^2^ = 50.7%).

Furthermore, Simon et al. [21] and Speer et al. [25] each studied separate cohorts of patients with and without anti-CD20 therapies. From these, drastically poorer responses could be observed in the anti-CD20 cohorts with 6 of 33 (18.2%) and 0 of 8 (0.0%) of anti-CD20 patients, respectively, studied by Simon et al. and Speer et al. seroconverting after the booster dose, compared to 26 of 33 (78.8%) and 12 of 13 (92.3%) patients without anti-CD20 therapy.

We further performed subgroup analysis according to the underlying IMIDs. The subgroup effect was significant (*p*-value = 0.0286). As many studies included a mixed of IMIDs, this subgroup analysis was limited. In regard to the increasing risk of seroconversion, the cohort including only MS patients had the lowest risk of 0.06 (95% CI: 0.01–0.34), followed by RA including 76 patients from three cohorts with a pooled risk of 0.51 (95% CI: 0.12–0.89), ANCA vasculitis including 36 patients from two cohorts with a pooled risk of 0.53 (95% CI: 0.37–0.68). The subgroup for SLE consisted of eight patients who all seroconverted, leading to a subgroup risk with random effects of 0.94 (95% CI: 0.50–1.00). It is worth noting that the SLE patients studied by Assawasaksakul et al. [35] were not on anti-CD20 therapy, which may have contributed to the higher response rate, rather than the IMID itself. In contrast, the 16 MS patients studied by Achtnichts et al. [30] were all on anti-CD20 therapies, ocrelizumab or rituximab. Significant heterogeneity was noted in all subgroups except for ANCA vasculitis.

Subgroup analysis by vaccine type was conducted by pooling cohorts which received only one type of COVID-19 vaccine as a booster (test of subgroup differences, *p*-value = 0.0049). Cohorts which included only mRNA boosters had a seroconversion rate of 0.34 (95% CI: 0.16–0.58), higher than viral vector boosters with 0.22 (95% CI: 0.10–0.41). A significant number of cohorts received a mix of mRNA and non-mRNA vaccines, making it difficult in the current analysis to isolate the effect of vaccine type on seroconversion. Subgroup analysis by age did not demonstrate significant differences (*p*-value = 0.0571).

### 3.3. Rise in Antibody Levels after Booster Dose

As we anticipated there to be significant heterogeneity in the measurement and reporting of antibody levels, such as in the assay used and specific antibody measured, a systematic review without a meta-analysis approach was used to qualitatively assess antibody levels reported in Table 3.

Across all studies, booster doses resulted in a significant rise in antibody level. This ranged from an increase of 2.19-fold [23] to over 6250-fold [34]. In the study by Connolly et al., the median antibody level measured by the Roche Elecsys anti-RBD pan-Ig rose from less than 0.4 U/mL to over 2500 U/mL.

### 3.4. Increased Seroprotection against COVID-19 Variants of Significance

Mallory et al. [33] performed assays against COVID-19 variants of significance, Alpha, Beta, Delta and Omicron before and after the booster dose. A functional hACE2 receptor binding inhibition assay was performed before and after the booster dose, demonstrating respective rises in inhibition of 54.4-fold (ancestral), 21.9-fold (Alpha), 24.5-fold (Beta), 24.4-fold (Delta), and 20.1-fold (Omicron). Additionally, anti-rS IgG activity assays performed demonstrated marked rises in antibody response against all variants of significance.

Rituximab therapy appeared to be the most notable factor impairing the ability of IMID patients to mount an immune response against variants such as Delta. Speer et al. [25] found that the booster dose elicited Delta-neutralising activity in 12 of 13 (92%) without rituximab therapy. None of the eight patients on rituximab therapy demonstrated this.

Hadjadj et al. [27] studied the rise in neutralising antibodies against Alpha and Delta variants and found that neutralising activity increased in patients receiving methotrexate and other immunosuppressive drugs including steroids, csDMARDs, and biologics, except for rituximab. The cohort receiving rituximab did not exhibit increased neutralising activity against Alpha and Delta variants after the booster dose. Jyssum et al. [24] similarly found that rituximab-receiving patients did not exhibit significant seroconversion after the booster dose, but all had induced CD4 and CD8 T-cell responses to the Delta variant.

### 3.5. Reactogenicity and Adverse Events after the Booster Dose

Local and systemic adverse events following the booster dose was sought from each study. Furthermore, the incidence and severity of adverse events after the booster were compared to that after the first and/or second vaccine dose if reported by the authors. The largest study, including 211 IMID patients, by Dreyer-Alster et al. [32], reported 115 (54.5%) experiencing an adverse event, with fever/chills, fatigue, and injection site pain being the most common at 52 (24.6%), 50 (23.7%), and 46 (21.8%) occurrences, respectively. Importantly, seven patients (3.3%) reported an acute relapse of MS occurring a median (range) of 34 (14–67) days after receiving the booster dose.

Jyssum et al. [24] noted that the frequency of adverse events was comparable in IMID patients after the second (32 of 67, 48%) and booster (19 of 45, 42%) dose of mRNA vaccines. The frequency of specific adverse events was comparable after the second and booster doses, except for bleeding and bruises which were more frequent after the booster than the second dose. While disease flares were observed, they were rare and comparable in incidence to the primary vaccination series. Among patients who received a booster dose, 5 of 37 (14%), 3 of 39 (8%), and 7 of 45 (16%) reported IMID flares after the first, second, and booster doses, respectively.

Mallory et al. similarly found no major systemic adverse events after the booster dose of NVX-CoV2373, although the frequency of adverse events was higher after the booster dose (13.4% grade 3 and above) than primary series vaccinations (5.2% grade 3 and above). Two grade 4 local adverse events, pain and tenderness, were reported by one patient receiving a booster dose, while none did after the primary series. The case series by Schmiedeberg et al. [22] and Assawasaksakul et al. [35] similarly only reported transient local adverse events such as pain and systemic adverse events such as fatigue and fever.

While no serious adverse events such myocarditis or death were reported, it should be keenly noted that the sample populations receiving booster doses studied in this review are generally small.

### 3.6. Publication Bias, Influence and Sensitivity Analysis

Publication bias was not suggested when assessed visually using a trim-and-fill funnel plot (Figure S1) and quantitatively with Egger’s test (Figure S2). Leave-one-out analysis (Figure S3) and repeating analysis with either the fixed effects model (Figure S4) or random effects model with Hartung–Knapp adjustment (Figure S5) did not show significant changes in the overall results. Only two studies by Schmiedeberg et al. [22] and Jyssum et al. [24] were identified as outliers (Figure S6).

## 4. Discussion

### 4.1. Efficacy, Necessity, and Safety of a Booster Dose

Our systematic review and meta-analysis has demonstrated that booster doses of COVID-19 vaccines, whether heterologous or homologous, are effective in affording seroprotection in patients who were non-responders to the primary series.

Around the world, it has been demonstrated that the vaccine-induced immune response wanes over time, leading to experts to advocate the necessity of a booster dose [38,39]. Hadjadj et al. [27] found that in patients in the cohort that did not receive a booster dose, Alpha and Delta neutralising activity fell by 3.5- and 5-fold, respectively, though anti-spike IgG levels were maintained. While booster doses have, as of current evidence and to the best of our knowledge, not been associated with significant adverse events such as disease flares and mortality, healthcare professionals and policymakers should remain watchful of developing evidence.

### 4.2. Factors Predicting Non-Response after a Booster Dose

It has been established that anti-CD20 therapies significantly impair seroconversion rate and depress antibody levels even in those who do seroconvert. Our subgroup analysis of patients on anti-CD20 therapy versus those without concurred with this with a significant test for subgroup differences (*p*-value < 0.0001). Jyssum et al. [24] further determined that patients with a longer duration from last rituximab infusion had better immune responses to vaccines, with a median of 267 days (IQR: 222–324) from last infusion in responders compared to 107 days (IQR: 80–152) in non-responders. This suggests that timing of vaccine doses further from last rituximab infusion to allow time for immune reconstitution may be promising. While withholding immunosuppressive therapy peri-vaccination or timing of vaccinations a set time from last immunosuppressive therapy has been suggested, the evidence for this is not definitive [40,41,42].

Studies of one or two doses of mRNA vaccines demonstrated varying immune response in patients on ocrelizumab [43,44,45] and depressed response in those on belimumab [46]. However, amongst patients studied in this review, rituximab was the main anti-CD20 therapy used and thus we are unable to conclude if other anti-CD20 therapies, such as ocrelizumab, or BLyS-specific inhibitors, such as belimumab, exhibit a similar impaired response. Other than rituximab treatment, Schell et al. [23] determined that anti-TNF therapy may be associated with poorer response.

Immune cell counts, including B-cell, CD4, and CD8 T-cell counts, have been postulated to correlate with immunocompromised patients’ ability to mount a response. Sidler et al. [26] elucidated that patients with higher CD19 counts at baseline may correlated to improved seroconversion, though statistical significance may be limited by a small sample size. Jyssum et al. [24] also found CD19 counts to correlate significantly with response to the second dose.

Patients mounting no response or an undetectable response to the primary vaccine series may be less likely to seroconvert, even if given a booster dose. Connolly et al. [34] found that 80% of the IMID patients who exhibited no detectable response after completion of the primary series seroconverted after the booster. While a majority still seroconverted, this is in contrast to IMID patients with a low-positive response after the primary series, 100% of whom seroconverted.

Another point of optimizing seroconversion rates potentially lies in the choice of COVID-19 vaccine administered as a booster. Schell et al. [23] found that antibody levels were significantly higher in those who received three Moderna doses (Median: 94 [IQR 38–170]) compared to those who received three Pfizer doses (Median: 62 [IQR 31–96]), *p*-value = 0.047. The idea of heterologous COVID-19 vaccines has been explored in immunocompetent people [47,48,49] as well as other immunocompromised patients such as solid organ transplant recipients [50], but we were unable to elucidate definite evidence of its efficacy in IMID patients.

### 4.3. Limitations of Review

Firstly, most studies included were observational studies which may not control adequately for factors such as age, disease type, and type of COVID-19 vaccine. To address this, we undertook subgroup analysis according to these factors identified, which suggests there may be significant effect moderation due to vaccine and disease type but not age. IMID patients included in this meta-analysis also were on various immunosuppressive treatments, contributing to heterogeneity in the overall analysis. To address this, we performed subgroup analysis by anti-CD20-containing and non-anti-CD20 regimens, which resulted in little remaining heterogeneity.

Secondly, there is a paucity of published evidence which limited the analysis. Future studies which report data of patients stratified by other treatment factors, such as steroid dose, would allow us to elicit factors predicting poor response to COVID-19 vaccines. Researchers may also seek to further assess the efficacy of heterologous, especially non-mRNA vaccines, as a booster dose, as most patients included in this meta-analysis received the mRNA vaccines BNT162b2 and mRNA-1273.

## 5. Conclusions

COVID-19 vaccination is one of the foremost preventive strategies against symptomatic and severe infection with COVID-19 in patients receiving immunosuppressive therapy. While non-response to vaccination poses a public health challenge, strategies to mitigate this, such as booster vaccination in non-responders, have been explored. Our systematic review and meta-analysis has shown that booster doses have the propensity to elicit seroconversion in 47% of IMID patients who were non-responders to the primary series of COVID-19 vaccination and with strengthened neutralizing responses to COVID-19 and its emergent variants of significance, including Delta and Omicron. Furthermore, it has not been associated with an increased frequency or severity of adverse events compared to the primary COVID-19 vaccine series.

## Figures and Tables

**Table 1:** Characteristics of included studies.

Study	Study Design	Primary Series Received	Booster Vaccine	IMIDs	Treatment Received	Seroconversion Threshold	Age *, Years	Days * between Booster Dose and Primary Series
Simon et al. [21]	Observational	BNT162b2 or AZD1222	BNT162b2 or AZD1222	66 total:	5 TNF	SARS-CoV-2 spike protein IgG OD450nm > 1.1	Mean (SD): 63.3 (14)	RTX: 93 Non-RTX: 69
					2 IL-17			
					1 IL-6			
					1 IL-1			
					33 RTX			
				30 RA	5 CD80/86			
				4 SA	22 csDMARD			
				13 CTD	30 steroids			
				14 vasculitis	7 JAKi			
				5 others	1 Integrin α4β7			
Schmiedeberg et al. [22]	Observational	BNT162b2	BNT162b2	17 RA	5 combined csDMARD and biologics	SARS-CoV-2 S1 IgG > 133U/mL	Not reported	Not reported
					1 csDMARD monotherapy			
					3 biologic monotherapy			
					3 JAKi monotherapy			
Schell et al. [23]	Observational	BNT162b2 or mRNA-1273	BNT62b2 or mRNA-1273	85 total	3 ASA	Anti-spike IgG, cutoff not reported	48 (38–60)	149 (132–167)
				55 CD	21 vedolizumab monotherapy			
					6 thiopurine			
					31 anti-TNF mono			
					12 anti-TNF combination			
				30 UC	9 ustekinumab monotherapy or combination			
					2 tofacitinib monotherapy			
					1 steroids			
Jyssum et al. [24]	Observational	BNT162b2 or mRNA-1273	BNT162b2 or mRNA-1273	49 RA	16 RTX monotherapy	anti-RBD > 70 AU/mL	62 (56–67)	Not reported
					5 steroids			
					22 MTX			
Speer et al. [25]	Observational	BNT162b2	BNT162b2	21 ANCA vasculitis	4 only steroids	Viral neutralisation > 30%	71 (59–74)	103
					9 Azathioprine or MMF			
					8 RTX ± azathioprine or MMF ± steroids			
Sidler et al. [26]	Observational	BNT162b2	BNT162b2 or mRNA-1273	Not reported	Anti-CD20 therapies (RTX or ocrelizumab)	Anti-SARS-CoV-2 IgG s/c ratio > 1.1	66 (50–72) (Anti-S1 negative patients) 58 (43–71) (Anti-S1 positive patients)	5 months
Hadjadj et al. [27]	Observational	BNT162b2	BNT162b2	56 total:	19 MTX	anti-SARS-CoV-2 IgG > 1.1 BAU/mL, IgA > 0.2 BAU/mL	52 (37.8–66.3)	102
					5 azathioprine			
				18 vasculitis	12 MMF			
				15 SLE	3 CYC			
				7 SS	6 anti-TNF			
				2 Sjogren’s	22 RTX			
				5 myositis	3 tocilizumab			
				3 arthritis	1 belimumab			
				6 others	15 HCQ			
Yang et al. [28]	Observational	BNT162b2, mRNA-1273 or Ad26.COV2.S	BNT162b2, mRNA-1273 or Ad26.COV2.S	35 with various IMIDs	8 anti-CD20 antibodies	Anti-S1 IgG OD ratio > 1.1	55 (38–63)	Not reported
					2 S1P modulators			
					9 MMF			
					10 steroids			
					1 untreated			
Bonelli et al. [29]	Observational	BNT162b2 or mRNA-1273	BNT162b2, mRNA-1273 or AZD1222	55 total:	10 MTX	Anti-RBD IgG > 0.8 BAU/mL	Patients receiving viral vector vaccines: 60.9 Patients receiving mRNA vaccines: 58.9	Not reported
					6 MMF			
				21 arthritis	5 azathioprine			
				16 CTD	4 leflunamide			
				8 vasculitis	4 HCQ			
				6 MS	2 Ig therapy			
				4 IgG4	15 steroids			
Achtnichts et al. [30]	Observational	BNT162b2 or mRNA-1273	BNT162b2 or mRNA-1273	16 MS	16 RTX or ocrelizumab	Anti-RBD > 100 AU/mL	Mean (SD): 51 (12.3)	104.3 (Range: 46–211)
Madelon et al. [31]	Observational	BNT162b2 or mRNA-1273	BNT162b2 or mRNA-1273	20 MS	20 ocrelizumab	Not reported	45.8 (37.8–53.3)	187 (156–203)
Dreyer-Alster et al. [32]	Observational	BNT162b2	BNT162b2	211 MS in total	53 untreated	Anti-S1 >35.2 BAU/ml	18–55 years: 121 > 55 years: 90	66 (54–84)
					6 beta-interferons			
					2 glatiramer acetate			
					19 teriflunomide			
				35 MS with serology data available	9 dimethyl fumarate			
					17 natalizumab			
					25 fingolimod			
					65 ocrelizumab			
				211 MS with safety data available	4 alemtuzumab			
					7 cladribine			
					1 RTX			
					3 intravenous immunoglobulins			
Mallory et al. [33]	Randomised trial	NVX-CoV2373	NVX-CoV2373	Various	Various	Inhibition concentration > 50%	57	189
Connolly et al. [34]	Case series	BNT162b2, mRNA-1273 or Ad26.COV2.S	BNT162b2, mRNA-1273 or Ad26.COV2.S	18 total:	Various	Anti-RBD > 500 U/mL	55 (44–65)	77
				1 MS				
				2 IBD				
				6 myositis				
				1 SLE				
				2 autoimmune hepatitis				
				3 arthritis				
				1 sarcoid				
				2 others				
Assawasaksakul et al. [35]	Case series	CoronaVac	BNT162b2 or AZD1222	8 SLE	Azathioprine, cyclosporin, MMF, steroids, tacrolimus	Inhibition > 35%	28 (22–45.5)	92
Felten et al. [36]	Case series	BNT162b2, mRNA-1273 or AZD1222	BNT162b2 or mRNA-1273	10 total:	RTX-containing regimens	Anti-RBD IgG > 7.1 AU/mL	72 (67–79.5)	65
				9 RA				
				1 Stiff-person syndrome				
Kant et al. [37]	Case series	BNT162b2, mRNA-1273 or Ad26.COV2.S	BNT162b2, mRNA-1273 or Ad26.COV2.S	15 ANCA vasculitis	RTX-containing regimens	Anti-spike S1 IgG, cutoff not reported	69 (63.5–73)	Not reported

* Median (IQR) reported if available. Abbreviations: NR, non-responders after primary series; CTD, connective tissue disease; MS, multiple sclerosis; RA, rheumatoid arthritis; SA, spondyloarthritis; CD, Crohn’s disease; UC, ulcerative colitis; SLE, systemic lupus erythematosus; ANCA vasculitis, antineutrophil cytoplasmic antibody vasculitis; RTX, rituximab; MTX, methotrexate; JAKi, Janus kinase inhibitor; ASA, aminosalicylate.

**Table 2:** Subgroup analysis according to key categorical variables.

Variable	Cohorts	N in Subgroup	Pooled Risk with Random Effects (95% CI)	I^2^ (%)	Test for Subgroup Effect (*p*-Value)
**Treatment**					
Anti-CD20	9	226	0.25 (0.17–0.36)	50.7	< 0.0001
Non-anti-CD20	6	114	0.81 (0.72–0.87)	0.0	
**Disease**					
Only RA	3	76	0.51 (0.12–0.89)	90.0	0.0286
Only SLE	1	8	0.94 (0.50–1.00)	NIL	
Only ANCA vasculitis	2	36	0.53 (0.37–0.68)	0.0	
Only MS	1	16	0.06 (0.01–0.34)	NIL	
**Age**					
<50	1	8	0.94 (0.50–1.00)	NIL	0.0571
50–65	7	269	0.36 (0.20–0.56)	86.8	
>65	3	46	0.52 (0.38–0.66)	0.0	
**Vaccine type**					
Only mRNA	6	163	0.34 (0.16–0.58)	83.5	0.0049
Only viral vector	1	27	0.22 (0.10–0.41)	NIL	

Abbreviations: RR, risk ratio; CI, confidence interval.

**Table 3:** Antibody levels after completion of primary series of COVID-19 vaccination and a booster dose.

Study *	Antibody and Value Measured	Days Post Primary Series	Pre-Booster Titre (IQR) ^†^	Days Post Booster	Post-Booster Titre (IQR) ^†^	Fold Increase ^‡^
Schmiedeberg et al. [22]	Anti-S1 antibody level (U/mL)	-	19.5 (0.45–48)	14	2500 (798–2500)	128.21
Schell et al. [23]	Anti-RBD antibody titre	32 (29–34)	31 (16–61)	37 (32–47)	68 (32–147)	2.19
Jyssum et al. [24]	Anti-RBD antibody titre (AU/mL)	7–10	3 (2–18)	21	Rise: 0.96 (0.05–27.38)	-
Speer et al. [25]	Anti-S1 IgG index	103 (72–126)	0.1 (0.1–1.8)	21	5.6 (0.5–150)	56
Speer et al. [25]	Neutralising surrogate antibodies	103 (72–126)	9 (0–35)	21	56 (4–94)	6.2
Yang et al. [28]	Anti-S1 antibody (OD ratio)	14	1.2 (0.2–5.2)	At least 7	3.3 (1.0–7.9)	2.75
Yang et al. [28]	ACE2 blocking (%)	14	0.0 (0.0–10.2)	At least 7	9.0 (0.0–42.5)	NA
Madelon et al. [31]	Anti-RBD antibody titre (U/mL)	-	GMT: 3.5	30	GMT: 57.9	16.5
Dreyer-Alster et al. (Cladribine) [32]	Anti-S1 antibody titre (BAU/mL)	At least 6 months	GMT: 686.3	0–3 months	GMT: 2345.6	3.42
Dreyer-Alster et al. (Glatiramer acetate) [32]	Anti-S1 antibody titre (BAU/mL)	At least 6 months	GMT: 581.9	0–3 months	GMT: 2530.1	4.35
Dreyer-Alster et al. (Diroximelfumarate) [32]	Anti-S1 antibody titre (BAU/mL)	At least 6 months	GMT: 335.7	0–3 months	GMT: 5830.4	17.37
Dreyer-Alster et al. (Immunoglobulins) [32]	Anti-S1 antibody titre (BAU/mL)	At least 6 months	GMT: 145.8	0–3 months	GMT: 5077.4	34.82
Dreyer-Alster et al. (Natalizumab) [32]	Anti-S1 antibody titre (BAU/mL)	At least 6 months	GMT: 286.6	0–3 months	GMT: 2161.4	7.54
Dreyer-Alster et al. (Dimethyl fumarate) [32]	Anti-S1 antibody titre (BAU/mL)	At least 6 months	GMT: 181.8	0–3 months	GMT: 2255.6	12.41
Dreyer-Alster et al. (Teriflunomide) [32]	Anti-S1 antibody titre (BAU/mL)	At least 6 months	GMT: 373.7	0–3 months	GMT: 2331.2	6.24
Mallory et al. [33]	Serum IgG against ancestral SARS-CoV-2 (EU)	14	43905	28	204367	4.65
Connolly et al. [34]	Anti-RBD antibody level (U/mL)	77	<0.4 (<0.4–222)	30 (27–36)	2500 (885–2500)	>6250
Assawasaksakul et al. [35]	Anti-RBD antibody level (U/mL)	-	83.3 (31.6–341.6)	14	19,986 (15,079–59,735)	239.93

* Includes data from all participants irrespective of serological status after primary series. ^†^ Median and interquartile ranges reported unless otherwise stated. ^‡^ Interquartile range unless otherwise stated. Abbreviations: RBD, receptor-binding domain; GMT, geometric mean titre.

## Data Availability

No new data were created or analyzed in this study. Data sharing is not applicable to this article.

## Supplementary Materials

The following supporting information can be downloaded at: https://www.mdpi.com/article/10.3390/vaccines10050668/s1, Table S1: Search strategy; Table S2: Comparison of characteristics of studies included in meta-analysis; Table S3: Quality assessment of included cohort studies using the Joanna Brigg’s Institute Critical Appraisal tool; Table S4: Quality assessment of included case series using the Joanna Brigg’s Institute Critical Appraisal tool; Table S5: Quality assessment of included randomised-controlled trials using the Joanna Brigg’s Institute Critical Appraisal tool; Figure S1: Trim-and-fill funnel plot with imputation of potentially missing studies; Figure S2: Linear regression test of funnel plot asymmetry; Figure S3: Leave-one-out analysis; Figure S4: Rate of seroconversion after administration of a booster dose in non-responders to a primary series of COVID-19 vaccination using the fixed effects model; Figure S5: Rate of seroconversion after administration of a booster dose in non-responders to a primary series of COVID-19 vaccination using the random effects model with Hartung-Knapp adjustment; Figure S6: Identification and exclusion of outliers.
