# Supplementary material for: Booster COVID-19 Vaccines for Immune-Mediated Inflammatory Disease Patients: A Systematic Review and Meta-Analysis of Efficacy and Safety
Source: Vaccines (Basel). 2022 Apr 22;10(5):668. doi: 10.3390/vaccines10050668 (PMC9144569; doi:10.3390/vaccines10050668)
Supplement: Supplementary file 1 [file vaccines-10-00668-s001.zip › vaccines-1656056-supplementary.pdf]

Supplementary Material Contents

Table S1: Search strategy
Table S2: Comparison of characteristics of studies included in meta-analysis
Table S3: Quality assessment of included cohort studies using the Joanna Brigg's Institute Critical Appraisal tool
Table S4: Quality assessment of included case series using the Joanna Brigg's Institute Critical Appraisal tool
Table S5: Quality assessment of included randomized-controlled trials using the Joanna Brigg's Institute Critical Appraisal tool
Figure S1: Trim-and-fill funnel plot with imputation of potentially missing studies
Figure S2: Linear regression test of funnel plot asymmetry
Figure S3: Leave-one-out analysis
Figure S4: Rate of seroconversion after administration of a booster dose in non-responders to a primary series of COVID-19 vaccination using the fixed effects model
Figure S5: Rate of seroconversion after administration of a booster dose in non-responders to a primary series of COVID-19 vaccination using the random effects model with Hartung-Knapp adjustment
Figure S6: Identification and exclusion of outliers

Table S1. Search strategy.

Database	Search terms	Results
PubMed	(covid OR sars) AND (vacc* OR immunis*) AND (immunodef* or immunosupp* or immunocomp* or autoimmune or autoinflammatory or rheum* or inflammatory bowel or Crohn's or ulcerative or psoria* or ankylosing or spondylitis or spondyloarthritis or multiple sclerosis or lupus or vasculitis or myositis or methotrexate or azathioprine or steroid or janus kinase or rituximab or anti cd20 or mycophenolate or interleukin or csdmard or dmard or tacrolimus or cyclosporin or ocrelizumab or hydroxychloroquine)<br><br>Search limits: 1 January 2021 to 28 February 2022	3,029
EMBASE	(covid OR sars) AND (vacc* OR immunis*) AND (immunodef* OR 'immunodeficiency'/exp OR immunosupp* OR 'immunosuppression'/exp OR immunocomp* OR immunocompromised OR autoimmune OR autoinflammatory OR rheumatic OR (inflammatory AND bowel) OR crohn OR ulcerative OR 'psoriasis'/exp OR (ankylosing AND spondylitis) OR spondyloarthritis OR (multiple AND sclerosis) OR lupus OR vasculitis OR myositis OR methotrexate OR azathioprine OR steroid OR (janus AND kinase) OR rituximab OR (anti AND cd20) OR mycophenolate OR interleukin OR csdmard OR dmard OR tacrolimus OR cyclosporin OR ocrelizumab OR hydroxychloroquine) NOT [medline]/lim<br><br>Search limits: 1 January 2021 to 28 February 2022	2,139
CENTRAL	(covid OR sars) AND (vacc* OR immunis*) AND (immunodef* or immunosupp* or immunocomp* or autoimmune or autoinflammatory or rheum* or inflammatory bowel or Crohn's or ulcerative or psoria* or ankylosing or spondylitis or spondyloarthritis or multiple sclerosis or lupus or vasculitis or myositis or methotrexate or azathioprine or steroid or janus kinase or rituximab or anti cd20 or mycophenolate or interleukin or csdmard or dmard or tacrolimus or cyclosporin or ocrelizumab or hydroxychloroquine)<br><br>Search limits: 1 January 2021 to 28 February 2022	104
Web of Science	(covid OR sars) AND (vacc* OR immunis*) AND (immunodef* or immunosupp* or immunocomp* or autoimmune or autoinflammatory or rheum* or inflammatory bowel or Crohn's or ulcerative or psoria* or ankylosing or spondylitis or spondyloarthritis or multiple sclerosis or lupus or vasculitis or myositis or methotrexate or azathioprine or steroid or janus kinase or rituximab or anti cd20 or mycophenolate or interleukin or csdmard or dmard or tacrolimus or cyclosporin or ocrelizumab or hydroxychloroquine)<br><br>Search limits: 1 January 2021 to 28 February 2022	2,314

Table S2. Comparison of characteristics of studies included in meta-analysis.

Study	Vaccine type		Diseases					Treatment		Vaccine response
	mRNA	Viral vector	RA	MS	SLE	Vasculitis	Others	Anti-CD20	Other DMARDs	Antibody assessed
Achtnichts	+ (16)			+ (16)				+ (16)		Anti-RBD
Jyssum	+ (49)		+ (49)					+ (49)		Anti-RBD
Sidler	+ (32)						+ (32)	+ (32)		Anti-S1
Bonelli	+ (28)	+ (27)		+ (6)		+ (8)	+ (41)	+ (55)		Anti-RBD
Kant	+ (14)	+ (1)				+ (15)		+ (15)		Anti-S1
Simon	+	+	+ (30)			+ (14)	+ (22)	+ (33)	+ (33)	Anti-S1
Felten	+ (10)		+ (9)				+ (1)	+ (10)		Anti-S1
Speer	+ (21)					+ (21)		+ (8)	+ (13)	Anti-S1
Yang	+	+					+ (35)	+ (8)	+ (27)	Anti-S1
Connolly	+ (12)	+ (6)		+ (1)	+ (1)		+ (14)		+ (16)	Anti-RBD
Schmiedeberg	+ (17)		+ (17)						+ (17)	Anti-S1
Assawasaksakul	+ (7)	+ (1)			+ (8)				+ (8)	Anti-RBD

Values in parentheses indicate number of patients, where available.

Table S3. Quality assessment of included cohort studies using the Joanna Brigg's Institute Critical Appraisal tool.

Study	1	2	3	4	5	6	7	8	9	10	11	Overall
Bonelli	NA	NA	Y	Y	N	Y	Y	Y	Y	U	Y	Include
Sidler	NA	NA	Y	Y	Y	Y	Y	Y	Y	U	Y	Include
Simon	NA	NA	Y	Y	Y	Y	Y	Y	Y	NA	Y	Include
Schmiedeberg	NA	NA	Y	Y	N	Y	Y	Y	Y	NA	Y	Include
Schell	NA	NA	Y	Y	N	Y	Y	Y	Y	U	Y	Include
Yang	NA	NA	Y	Y	Y	Y	Y	Y	Y	U	Y	Include
Speer	NA	NA	Y	Y	Y	Y	Y	Y	Y	NA	Y	Include
Hadjadj	NA	NA	Y	Y	Y	Y	Y	Y	Y	U	Y	Include
Jyssum	NA	NA	Y	Y	Y	Y	Y	Y	Y	Y	Y	Include
Achtnichts	NA	NA	Y	Y	Y	Y	Y	Y	Y	Y	Y	Include
Madelon	NA	NA	Y	Y	Y	Y	Y	Y	Y	NA	Y	Include
Dreyer-Alster	NA	NA	Y	Y	Y	Y	Y	Y	Y	NA	Y	Include

Checklist
1. Were the two groups similar and recruited from the same population?
2. Were the exposures measured similarly to assign people to both exposed and unexposed groups?
3. Was the exposure measured in a valid and reliable way?
4. Were confounding factors identified?
5. Were strategies to deal with confounding factors stated?
6. Were the groups/participants free of the outcome at the start of the study (or at the moment of exposure)?
7. Were the outcomes measured in a valid and reliable way?
8. Was the follow up time reported and sufficient to be long enough for outcomes to occur?
9. Was follow up complete, and if not, were the reasons to loss to follow up described and explored?
10. Were strategies to address incomplete follow up utilized?
11. Was appropriate statistical analysis used?

Legend:
Y – Yes
N – No
U – Unclear
NA – Not applicable

Table S4. Quality assessment of included case series using the Joanna Brigg's Institute Critical Appraisal tool.

Study	1	2	3	4	5	6	7	8	9	10	Overall
Connolly	Y	Y	Y	U	Y	Y	Y	Y	N	NA	Include
Assawasaksakul	Y	Y	Y	N	Y	Y	Y	Y	N	NA	Include
Felten	Y	Y	Y	U	Y	Y	Y	Y	N	NA	Include
Kant	Y	Y	Y	N	U	Y	Y	Y	N	NA	Include

Checklist
1. Were there clear criteria for inclusion in the case series?
2. Was the condition measured in a standard, reliable way for all participants included in the case series?
3. Were valid methods used for identification of the condition for all participants included in the case series?
4. Did the case series have consecutive inclusion of participants?
5. Did the case series have complete inclusion of participants?
6. Was there clear reporting of the demographics of the participants in the study?
7. Was there clear reporting of clinical information of the participants?
8. Were the outcomes or follow-up results of cases clearly reported?
9. Was there clear reporting of the presenting sites'/clinics' demographic information?
10. Was statistical analysis appropriate?

Legend:
Y – Yes
N – No
U – Unclear
NA – Not applicable

Table S5. Quality assessment of included randomised-controlled trials using the Joanna Brigg's Institute Critical Appraisal tool.

Study	1	2	3	4	5	6	7	8	9	10	11	12	13	Overall
Mallory	Y	Y	Y	N	Y	Y	Y	Y	Y	Y	Y	Y	Y	Include

Checklist
1. Was true randomization used for assignment of participants to treatment groups?
2. Was allocation to treatment groups concealed?
3. Were treatment groups similar at the baseline?
4. Were participants blind to treatment assignment?
5. Were those delivering treatment blind to treatment assignment?
6. Were outcomes assessors blind to treatment assignment?
7. Were treatment groups treated identically other than the intervention of interest?
8. Was follow up complete and if not, were differences between groups in terms of their follow up adequately described and analyzed?
9. Were participants analyzed in the groups to which they were randomized?
10. Were outcomes measured in the same way for treatment groups?
11. Were outcomes measured in a reliable way?
12. Was appropriate statistical analysis used?
13. Was the trial design appropriate, and any deviations from the standard RCT design (individual randomization, parallel groups) accounted for in the conduct and analysis of the trial?

Legend:
Y – Yes
N – No
U – Unclear
NA – Not applicable

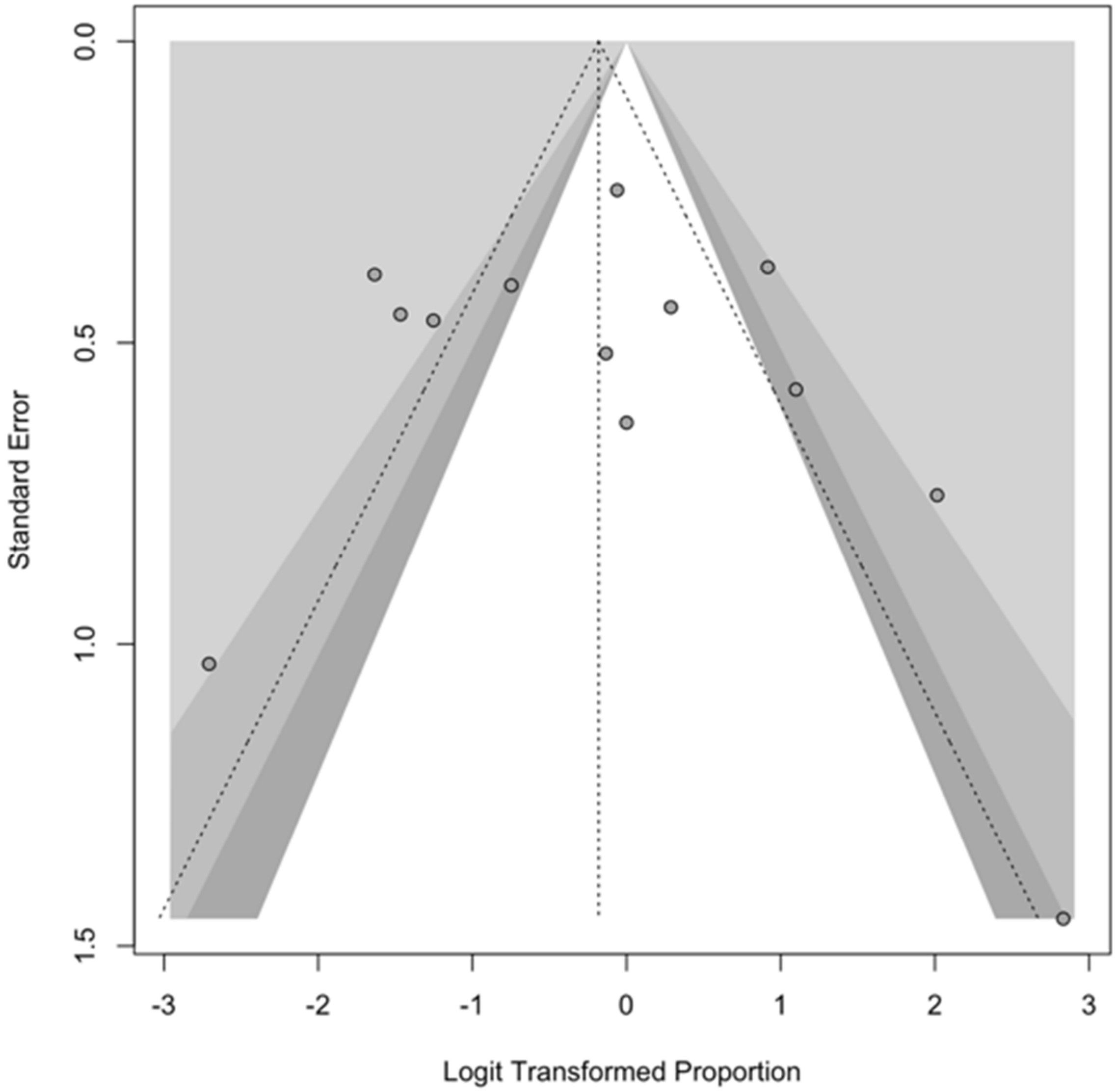

Figure S1. Trim-and-fill funnel plot with imputation of potentially missing studies.

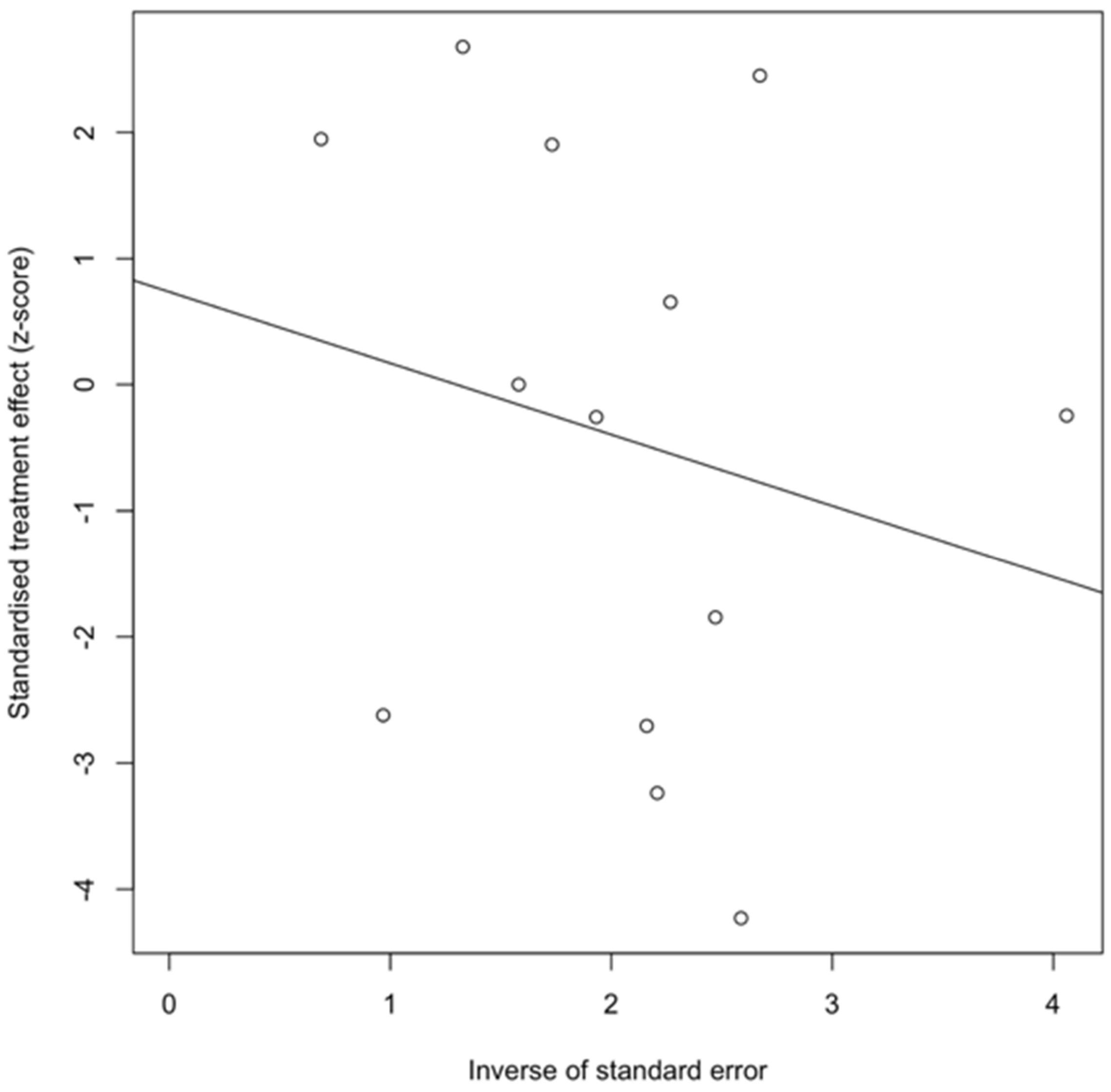

Figure S2. Linear regression test of funnel plot asymmetry.

Test result: t = 0.42, df = 11, p-value = 0.6849

Sample estimates:
bias se.bias intercept se.intercept
0.7342 1.7618 -0.5650 0.7970

Details:
- multiplicative residual heterogeneity variance (tau^2 = 5.6292)
- predictor: standard error
- weight: inverse variance
- reference: Egger et al. (1997), BMJ

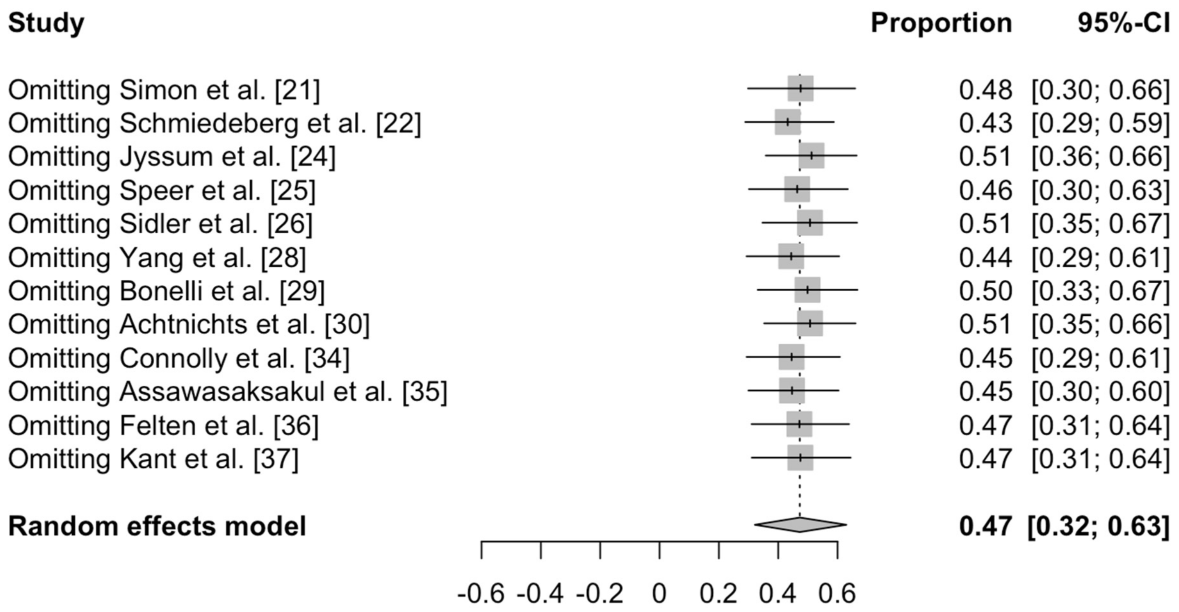

Figure S3. Leave-one-out analysis.

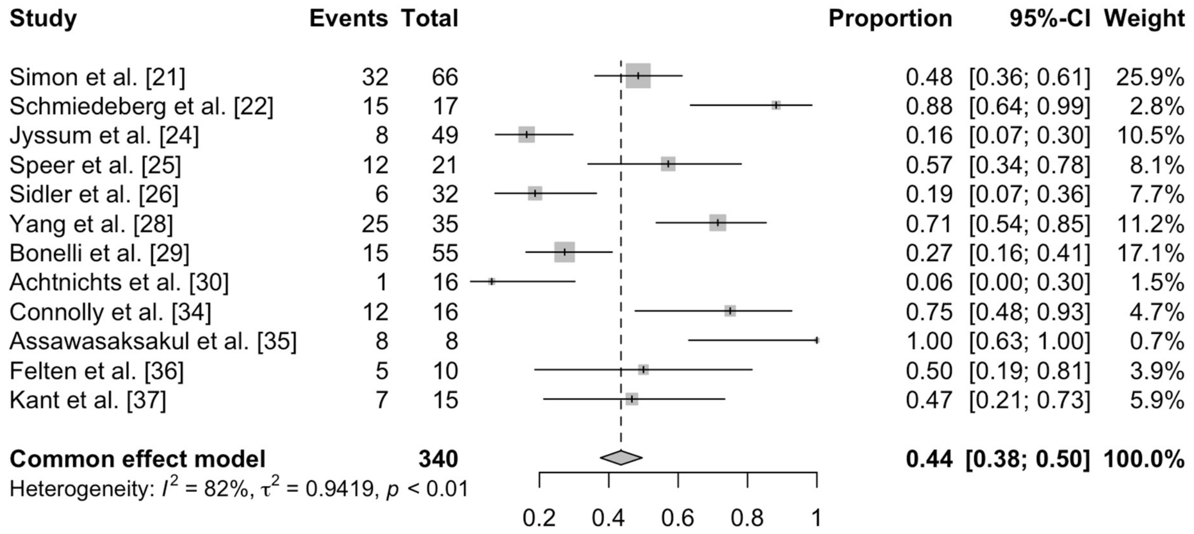

Figure S4. Rate of seroconversion after administration of a booster dose in non-responders to a primary series of COVID-19 vaccination using the fixed effects model.

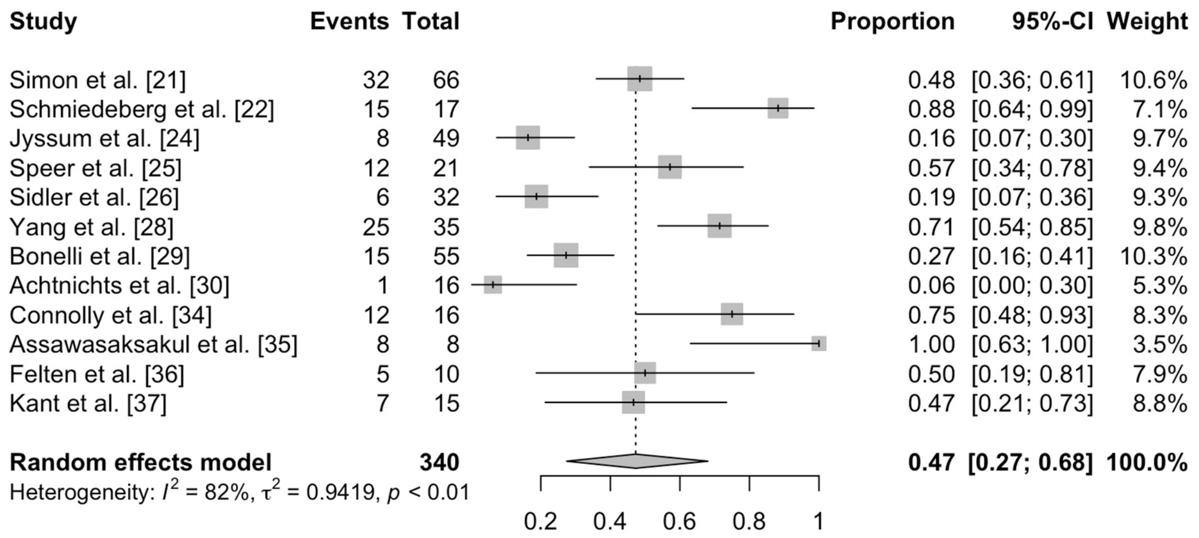

Figure S5. Rate of seroconversion after administration of a booster dose in non-responders to a primary series of COVID-19 vaccination using the random effects model with Hartung-Knapp adjustment.

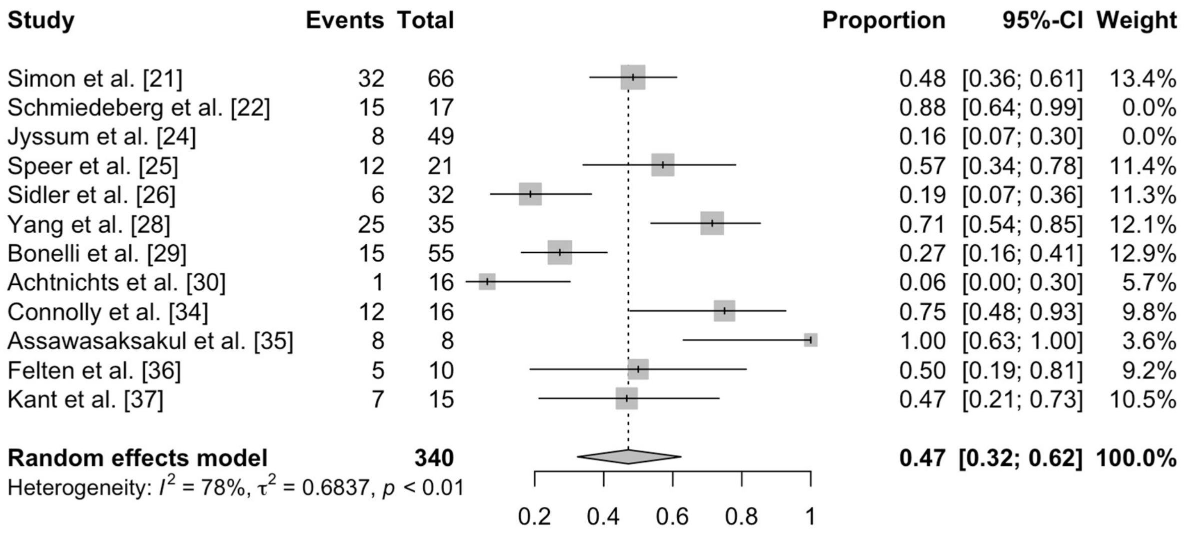

Figure S6. Identification and exclusion of outliers.
